# The Influence of Cervical Spine Angulation on Symptoms Associated With Wearing a Rigid Neck Collar

**DOI:** 10.1177/21514593211012391

**Published:** 2021-05-06

**Authors:** Taiwo D. Kelani, Annabelle Lee, Miny Walker, Louis J. Koizia, Melanie Dani, Michael B. Fertleman, Angela E. Kedgley

**Affiliations:** 1 Department of Bioengineering, Imperial College London, White City, London, UK; 2 Geriatric Medicine, Imperial College NHS Trust, London, UK; 3 Imaging Department, Imperial College Healthcare NHS Trust, London, UK; 4 Department of Bioengineering, Cutrale Perioperative and Ageing Group, Imperial College London, White City, London, UK

**Keywords:** c-spine, rigid collar, vertebral angulation, visual analogue scale, cervical lordosis

## Abstract

**Introduction:**

Rigid cervical spine collars can be used to maintain the position of the cervical spine following injury or surgery. However, they have been associated with difficulty swallowing, pressure sores and pain, particularly in older patients. We aimed to investigate the relationship between cervical spine angulation, a rigid neck collar and neck pain in healthy young and older adults.

**Methods:**

Twenty healthy young adults aged 25 ± 3 years and 17 healthy older adults aged 80 ± 8 years were tested. Magnetic resonance imaging scans of their cervical spines were taken before and after the rigid neck collar was worn for 1 hour. Measurement of vertebral angulation involved digitization of the scans and joint angle calculations using image processing software. Pain was quantified before and after the collar was worn, using a visual analogue scale.

**Results:**

Pain scores increased in the young group after the collar was worn (p = 0.001). The older group showed no difference in pain score after the collar was worn. Statistical tests showed no significant correlations between the change in cervical angles and the change in pain scores after the collar was worn.

**Discussion:**

The aging process may contribute to the changing distribution of subcutaneous tissue and increase risk of symptoms associated with wearing a collar. Oesophageal compression is not a result of collar use.

**Conclusion:**

There is no correlation between cervical spine vertebrae angulation and symptoms associated with wearing a neck collar. Generally, older individuals have greater cervical lordosis angles, and more straight and lordotic neck shapes. Older individuals may be more prone to skin-interface pressures from the neck collar than younger individuals.

## Introduction

Cervical spine (c-spine) immobilization is a commonly used treatment modality for stable or unstable vertebral fractures, ligamentous injury and after c-spine surgery. The use of a rigid cervical collar may maintain the head in position through interface forces between the orthosis and the patient’s neck.^1^ The rationale is to minimize tissue compression that may result in tissue injury or displacement of unstable injuries leading to secondary neurological injury.

Evidence for the efficacy of cervical immobilization before and after surgery is mixed. Some literature argues that pre-hospital cervical immobilization is not required for patients who are “alert, stable and co-operative”^2^ unless there is a decline in their consciousness^2^ and some believe that any movement after the first injury to the spinal cord will have little to no additional effect on the spinal cord.^3^ In contrast, there is evidence that recommends prehospital c-spine immobilization in patients older than 65 years.^4^ There is literature that indicates that in the cases of joint dislocation or instability, the application of a collar may lead to spinal cord damage.^5,6^ There are documented cases of worsened neurological injury due to the application of rigid orthoses causing hyperextension and fracture displacement, particularly in older patients with underlying degenerative spinal disease.^7,8^

**Figure 1.**
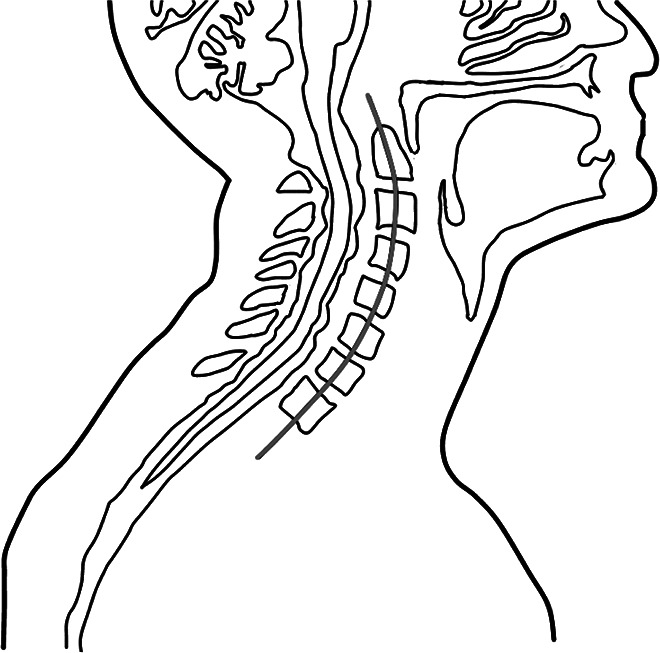
A schematic of the neck, showing the crescent shape formed by vertebrae in the c-spine (the lordotic curve) and surrounding structures.

The angulation of the c-spine is a key feature in patients with neck pain complaints. Vertebral angulation of the c-spine is the measurement of orientation and wedging of C1 to C7 vertebrae in relation to the head and trunk. The lordotic curve is the crescent shape formed by vertebrae found in the c-spine (Figure 1). The degree of lordotic curve in the c-spine is measured by the orientation of the foramen magnum and the wedging of the vertebral bodies and intervertebral discs.^9^ A study showed that 95% of men and 70% of women aged 60-65 years had at least 1 degenerative change to their c-spine and cervical lordosis increased with age in both gender groups.^10^ Patients who lack the lordotic curve in the c-spine and are treated with a rigid cervical orthosis have been shown to present with neck pain and stiffness in retrospective studies.^11^ However, prior studies that have measured vertebral angulation of the c-spine with a rigid cervical orthosis were performed on injured patients and did not quantify the degree of neck pain in individuals.^12^ In this case, the use of a psychometric measuring tool such as a visual analogue scale (VAS) would be beneficial. What is more, data collected from injured patients brings indistinction between pain from the injury and pain from the collar alone. The application of a neck collar alone can lead to increased temperature, humidity and cytokine concentration at skin-orthosis interface,^13^ which makes the skin increasingly susceptible to pressure ulcers.^13^ In addition to this, collars can prolong the duration of various stages of swallowing and may cause difficulties in swallowing^14^ that can contribute to problems with aspiration and further discomfort or pain.^12,15^ Cervical orthoses have also been found to exert pressure on the jugular venous system raising intracranial pressure.^8^

The purpose of this study was to evaluate the effect of a rigid cervical orthosis on neck pain, vertebral angulation, neck soft tissue and structures involved in swallowing.

## Methods

All participants provided written informed consent to take part in the study, which was approved by the local Research Ethics Committee. Exclusion criteria included those with current neck pain, known previous c-spine injury or any known medical condition affecting the spine. A post-hoc power analysis revealed that on the basis of the between-groups comparison effect size and within-groups comparison effect size, the statistical power for the young and elderly population groups were 0.8 and 0.8 respectively. Therefore, a total n of 20 in the young group and 20 in the older group would be needed to obtain statistical power at the recommended 80% level. However, due to the lack of participation, we were only able to obtain data from 17 participants in the older group.

Twenty healthy young adults (10 males and 10 females, 25 ± 3 years) and 17 healthy older individuals (10 males and 7 females, 80 ± 8 years) volunteered to participate. All participants completed a baseline VAS questionnaire to quantify their neck pain^16,17^ and then underwent a c-spine MRI scan (Maximize 1.5 T, MAGNETOM Area, Siemens). The images obtained were T2 sagittal CISS 3-D images with a slice thickness of 0.8 mm and each MRI examination was reviewed and reported by an experienced radiologist. Following this, participants were fitted with a rigid cervical orthosis (Miami J Collar, Össur, Reykjavik, Iceland); they were correctly sized and fitted by 2 trained and experienced clinicians. After 1 hour of wearing the collar, the participants completed a second VAS to quantify neck pain and a second MRI of the c-spine was performed while the participants were still wearing the collar. To complete the VAS participants were asked to mark their level of neck pain on a line between 2 endpoints 10 cm apart. The distance between 0 cm and the mark placed by the participant was rounded to the nearest whole number and was defined as the participant’s pain score.

**Figure 2.**
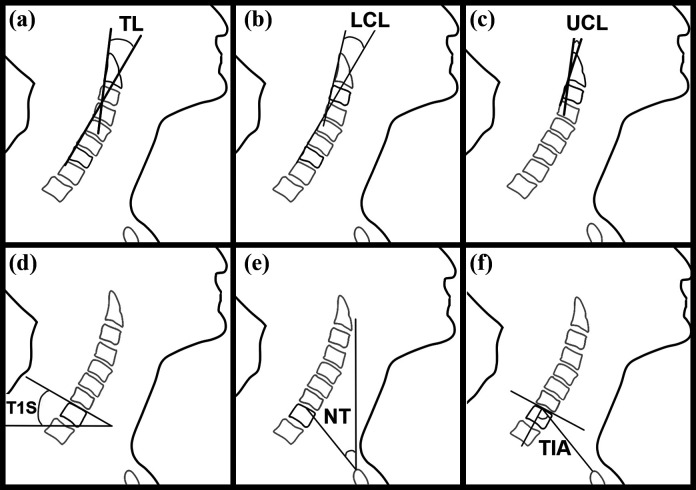
An Illustration of the cervical angles: (a) total lordosis (TL), (b) lower cervical lordosis (LCL), (c) upper cervical lordosis (UCL), (d) T1 slope (T1 S), (e) neck tilt (NT) and (f) thoracic inlet angle (TIA).

**Figure 3.**
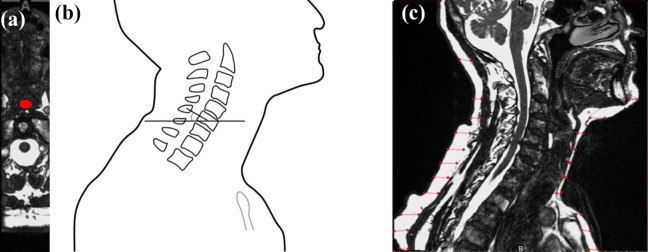
(a) The axial plane of the neck, displaying the area of the oesophagus. (b) An example of an individual vertebral angle (C6). (c) The midsagittal plane of the neck, displaying lines created to measure the thickness of the subcutaneous tissue.

**Figure 4.**
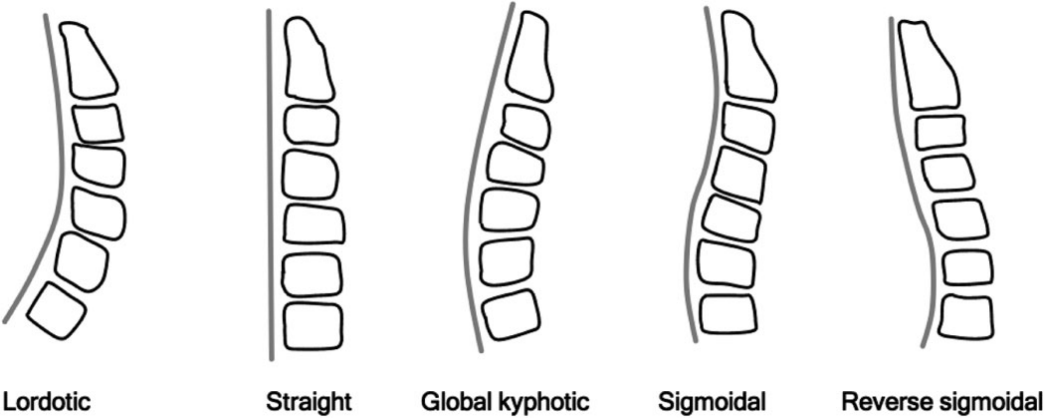
An illustration of the different neck subtype classifications.

The MRI scans were digitized (Mimics v. 17.0, Materialise, Leuven, Belgium); all measurements were performed on the midsagittal plane, unless stated otherwise. Total lordosis (TL), upper cervical lordosis (UCL), lower cervical lordosis (LCL), T1 slope (T1 S), neck tilt (NT) and thoracic inlet angle (TIA) were measured on each scan (Figure 2) and the individual vertebral angles were calculated for all vertebrae from C1 to T2. An example of the individual vertebral angle, C6 is illustrated in Figure 3b. Each of the participants were classified into a specific neck subtype group (Figure 4) to approximate a general sagittal alignment of the c-spine. The 5 neck subtypes were selected according to the modified Takeshima-Herbst subtype guidelines.^18^ The thickness of the subcutaneous tissue layer surrounding the c-spine in the mid-sagittal plane was measured for each of the scans (Figure 3c). The 2D area of the oesophagus was segmented and measured in the axial plane, from the level of C1 to the upper region of the sternum (Figure 3a).

Statistical analysis: All statistical analyses were performed on SPSS (IBM SPSS Statistics v. 24, IBM, Armonk, USA). A Shapiro-Wilk test was performed to confirm a normal distribution of the data. Descriptive statistics (mean ± standard deviation) were used for analysis of the vertebral angles, pain scores, oesophagus area and subcutaneous tissue measurements. The relationships between the measures were analyzed using factorial mixed ANOVA, paired samples t-test, Pearson’s correlation and independent t-test. For all outcomes, the statistical significance level was set to p < 0.05.

## Results

The Shapiro-Wilk test confirmed that the data was normally distributed. The paired samples t-test showed a significant increase in pain scores in the young group after the collar was worn (t = -3.541, df = 19, p = 0.001). The older group showed no significant difference between pre-and post-collar pain scores (t = -0.972, df = 16, p = 0.345).

**Figure 5.**
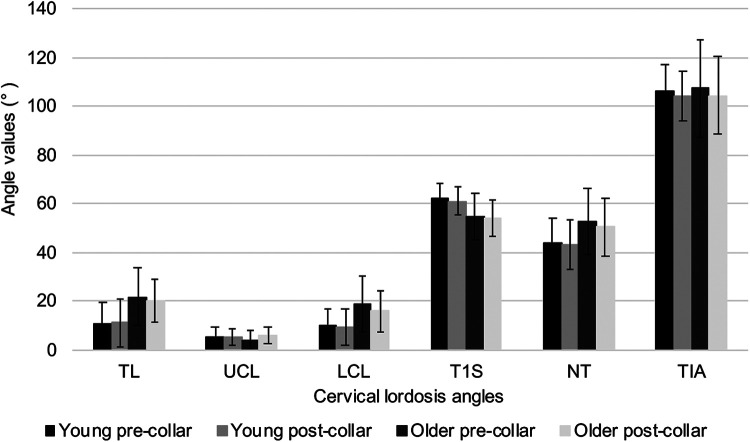
Mean (± 1 standard deviation) cervical lordosis angles of total lordosis (TL), upper cervical lordosis (UCL), lower cervical lordosis (LCL), T1 slope (T1 S), neck tilt (NT) and thoracic inlet angle (TIA) of the young and older groups, before and after the collar was worn.

The mean TL, UCL, LCL, NT and TIA angles were greater in the older group than in the young group, apart from T1 S, which was lower in the older group (Figure 5). Additionally, TL and LCL were the only angles that showed significantly greater values in the older group than the young group (p < 0.003). The remaining angles showed no differences when comparing the young and older groups and before and after the collar was worn. Pearson’s correlation test showed that the change in NT angle and TIA had a negative and non-significant correlation to the change in pain scores from before collar to after collar (r = -0.132, df = 35, p = 0.218), (r = -0.009, df = 35, p = 0.478), respectively. The remaining angles all showed positive non-significant correlations to the change in pain scores.

The baseline individual angles of the older group were greater than that of the young group (Table 1). The independent t-test showed a significant difference between young and older groups in the individual vertebral angles, C3 and C5 to T2 (p < 0.001). The remaining angles showed no significant differences between age groups and between before and after the collar was worn.

Forty percent of individuals in the young group were classified as the straight spine subtype, 20% were global kyphotic subtype, 20% were reverse sigmoidal subtype, 15% were sigmoidal subtype and 5% were lordotic subtype. Thirty-five percent of individuals in the older group were classified as the straight subtype, 20% were lordotic subtype, 15% were reverse sigmoidal subtype, 10% were global kyphotic subtype and 5% were sigmoidal subtype.

**Table 1.** Mean (± 1 Standard Deviation) of the Baseline Individual Vertebral Angles in the Young and Older Groups.

Baseline individual vertebral angles			
	Young group (°)	Older group (°)	Independent t-test p-value
C1	0.5 ± 11.3	0.2 ± 6.2	>0.050
C2	1.5 ± 7.0	-1.0 ± 4.6	>0.050
C3	-1.3 ± 6.1	-0.6 ± 3.5	0.030*
C4	-2.4 ± 7.1	-1.4 ± 3.8	0.928
C5	-2.2 ± 8.8	-1.3 ± 4.0	<0.001*
C6	-0.9 ± 9.7	-4.5 ± 2.9	<0.001*
C7	-1.3 ± 10.9	-5.0 ± 6.2	<0.001*
T1	-0.3 ± 9.5	-5.6 ± 6.5	<0.001*
T2	-0.9 ± 11.1	-3.8 ± 7.0	<0.001*

The subcutaneous tissue thickness was compressed more in the older participants (1.0 ± 2.7 mm) than in the young participants (0.1 ± 1.6 mm) at all observed vertebral levels (C1 to T2) while the collar was worn. In the young participant group, a decrease in subcutaneous tissue thickness was found posteriorly at the levels of C2 (p = 0.008) and C3 (p = 0.018) after the collar was worn. In the older participant group, a significant decrease in subcutaneous tissue thickness was found posteriorly at C1 (p = 0.049), C2 (p = 0.011), T2 (p = 0.032) and anteriorly at C6 (p = 0.023) after the collar was worn. The total pre-collar subcutaneous tissue thickness was greater at all vertebral levels in the older participants than the young.

An independent t-test showed no significant difference between the measurements of oesophagus areas in the 2 participant groups. Additionally, the oesophagus areas before and after the collar was worn showed no significant difference in either the young or older groups.

## Discussion

This study presents an analysis of the relationship between a rigid c-spine collar, vertebral angulation, neck pain and dimensions of structures surrounding the c-spine within a population of 37 healthy young and older adults.

The individual vertebral angles and the cervical lordosis angles showed no significant difference after the rigid collar was worn, suggesting that the collar does not affect the orientation of vertebrae. These findings are similar to a prior study^19^ that examined whether the degree of cervical lordosis changes after a course of spinal manipulative therapy for non-specific neck pain. Shilton et al^19^ found no significant change in cervical lordosis in patients after 4 weeks of cervical spinal manipulation,^19^ suggesting a weak relationship between spinal manipulative therapy and changes in the orientation of vertebrae.

There was a significant difference between the angles of the 2 age groups, whereby the majority of the older group’s average angles were greater than that of the young. This may perhaps be due to the large standard deviations of the vertebral angles of the older group. Other possible reasons for the increased angle in the older group could be related to ligamentous laxity or degenerative disc disease.^20^ Though this may need to be studied further with a greater sample size, the implication of the differences in c-spine alignment between age groups should be taken into consideration prior to fitting orthoses.

All the cervical lordosis angles showed non-significant correlations to the pain scores. The results of our study are comparable to a study carried out by Aşkin et al,^21^ who found no correlation between acute and chronic neck pain and the orientation of the cervical vertebrae. A shared limitation between the current study and that of Aşkin et al is a small sample size, which may contribute toward the lack of significant correlation.

Forty percent of the young group were classified as the straight spine subtype, 20% were global kyphotic subtype and 20% were reverse sigmoidal subtype. This suggests that the normal c-spine of young adults varies widely. This may be a reflection of the different activities to which a young person’s neck may be subject. Thirty-five percent of the older group were classified as the straight subtype and 20% were the lordotic subtype, suggesting that the normal c-spine of the older individuals is either lordotic or straight. This may be a result of compensatory changes in cervical alignment, which occurs to maintain forward head posture that may have been compromised by thoracic kyphosis, which is known to progress with age.^22,23^

As prominent skin-interface pressures are associated with inflammatory skin responses, the pressure distribution may be of use in collar design.^13^ Hence, the compressed areas of subcutaneous tissue in the young and older participants may provide important information for future design and development of rigid collars. In addition, the changing distribution of subcutaneous tissue as a result of the aging process may in part have an impact on increasing the risk of pressure sores developing in older patient groups.^24^

Though the collar did not significantly affect the area of the oesophagus, the oesophagus is only one of the structures involved in the process of swallowing. The effects on other structures involved in swallowing such as the vallecular space, mandible, tongue and hyoid are limited in literature. Therefore, further studies of these structures would provide a greater understanding of the effect of a rigid collar on swallowing. Although neck collars may extend the duration of various stages of swallowing and cause difficulties in swallowing,^14^ the results from the current study reject the idea that the rigid structure of the collar causes a direct constriction to the oesophagus. A dynamic imaging tool, such as a dynamic MRI or video fluoroscopy may be useful for future work in order to observe not only the changes in vertebral angulation, but the different stages of swallowing and the structures involved.

The interpretation of these results is subject to certain limitations. For instance, the time that the participants were wearing the collar. One hour is not clinically representative, as those who are injured would typically wear the collar consistently for a period of up to 8 weeks.^25^ However, to the authors knowledge this is one of few studies that investigate both pain and vertebral angulation with a rigid collar in healthy individuals. Future work should consider a longer time period for participants to wear the collar. It should be noted that there may be differences between different rigid collar models^26-28^ and as only 1 rigid collar model was investigated in this study, the results should be interpreted with caution. Though prior research has utilized static MRI measurements to assess dynamic function, the use of a dynamic modality such as dynamic MRI or videofluoroscopy may be more useful to observe the changes in vertebral angulation and the structures involved in the stages of swallowing. The use of the VAS as the only measurement for pain intensity is considered a limitation in this study, as though it is commonly used in clinical applications and has been validated in literature,^17,29-32^ its outputs may be influenced by factors unrelated to the desired target and thus are not universally accepted. This study was also limited by the small sample size of the older group. As previously stated, a sample size of 20 in the older group was required to obtain statistical power of 80%. However, due to low participation, only 17 participants were recruited in the older group.

## Conclusion

This study explored the relationships between neck pain, a rigid collar and vertebral joint angles in young and older adults. The results show that the rigid collar has no effect on the joint angles in the c-spine and there is no correlation between cervical lordosis angles and pain score. However, through the analysis of subcutaneous tissue thickness, a greater understanding has been gained of the particular areas in the neck that may be more prone to skin reactions due to a rigid collar. This brings opportunities for future work toward improved collar design to counter the reduced soft tissue observed in the older neck and hence aim to reduce risk of pressure sores arsing secondary to rigid collar immobilization.

The study shows there is no effect on oesophageal constriction in both young and older adults from rigid collar use. Moreover, as oesophageal compression is not a result of collar use and that the pain experienced is not due to the collar, this suggests that there are other causes for dysphagia during rigid cervical orthosis use. Future studies should target the effect of the collar on structures involved in swallowing and should include the use of dynamic MRI.
